# The impact of COVID-19 certification mandates on the number of cases of and hospitalizations with COVID-19 in the UK: A difference-in-differences analysis

**DOI:** 10.3389/fpubh.2023.1019223

**Published:** 2023-02-24

**Authors:** Kim López-Güell, Albert Prats-Uribe, Martí Català, Clara Prats, Jotun Hein, Daniel Prieto-Alhambra

**Affiliations:** ^1^Department of Statistics, University of Oxford, Oxford, United Kingdom; ^2^Centre for Statistics in Medicine, Nuffield Department of Orthopaedics, Rheumatology, and Musculoskeletal Sciences, University of Oxford, Oxford, United Kingdom; ^3^Escola Superior d'Agricultura de Barcelona, Campus del Baix Llobregat, Departament de Física i Enginyeria Nuclear, Universitat Politècnica de Catalunya, Barcelona, Spain

**Keywords:** covid certificate, covid passport, real-world data, observational studies, public health

## Abstract

**Background:**

Mandatory COVID-19 certification, showing proof of vaccination, negative test, or recent infection to access to public venues, was introduced at different times in the four countries of the UK. We aim to study its effects on the incidence of cases and hospital admissions.

**Methods:**

We performed Negative binomial segmented regression and ARIMA analyses for four countries (England, Northern Ireland, Scotland and Wales), and fitted Difference-in-Differences models to compare the latter three to England, as a negative control group, since it was the last country where COVID-19 certification was introduced. The main outcome was the weekly averaged incidence of COVID-19 cases and hospital admissions.

**Results:**

COVID-19 certification led to a decrease in the incidence of cases and hospital admissions in Northern Ireland, as well as in Wales during the second half of November. The same was seen for hospital admissions in Wales and Scotland during October. In Wales the incidence rate of cases in October already had a decreasing tendency, as well as in England, hence a particular impact of COVID-19 certification was less obvious. Method assumptions for the Difference-in-Differences analysis did not hold for Scotland. Additional NBSR and ARIMA models suggest similar results, while also accounting for correlation in the latter. The assessment of the effect in England itself leads one to believe that this intervention might not be strong enough for the Omicron variant, which was prevalent at the time of introduction of COVID-19 certification in the country.

**Conclusions:**

Mandatory COVID-19 certification reduced COVID-19 transmission and hospitalizations when Delta predominated in the UK, but lost efficacy when Omicron became the most common variant.

## Introduction

More than a year after the emergence of SARS-CoV-2, widespread transmission is arguably higher than ever. To date, 30th of January 2023, the virus has caused more than 22,200,000 confirmed cases, 990,000 hospital admissions and 217,000 deaths in the UK (1). All around western countries there has been a need to balance restrictions to fight the pandemic while controlling their impact on society.

The Delta variant of the virus became dominant soon after its arrival to the UK in the spring of 2021. The following November the UK reported the first confirmed cases of the Omicron variant. The UK Health Security Agency estimated the current prevalence of Omicron to be higher than 90% as of end-January, having quickly overcome Delta as the most common variant (2).

Since the emergence of the virus, various non-pharmaceutical interventions were introduced by several countries in Europe to fight against the COVID-19 pandemic. Their aim was to slow down the transmission by restricting mobility and social interactions, e.g., mass gathering measures. Several papers suggest some of them had an effect in reducing COVID-19 transmission (3–5). Recently, mandatory COVID-19 certification regulating access to public venues, nightclubs or cultural events was implemented in some countries, using proof of at least two doses of an approved vaccine, negative test (usually within the last 2 days) or a recovery certificate of a recent infection (usually within the previous 6 months) (6). Many voices have expressed concerns over its effectiveness and due to their potentially negative effects on the economy, for example in the hospitality sector. Some studies report increased vaccine uptake after its implementation (7, 8), but there is a lack of research on its potential impact in reducing incidence of COVID-19. It is possible the certification influenced the spread of the virus directly by restricting contact between individuals, or through acceleration of vaccination in the population.

The UK implemented COVID-19 certification during the second half of 2021, and each of its countries did it at different times. The certificate became mandatory to attend large events and nightclubs in October 2021 in Wales and Scotland, and in December 2021 in England. In addition to these events, a mandatory certificate also restricting access to cinemas, theaters and concert halls was implemented in November in Wales and Northern Ireland. More information on the application of the COVID-19 certificate in the different countries can be found in the *Methods* section. We took advantage of this natural experiment to study whether COVID-19 certification in the UK had an effect in reducing the incidence of COVID-19 cases and hospitalizations, considering the four countries separately. We use England as a negative control, since it was the last country where COVID-19 certification was introduced.

## Methods

### Data

Data on COVID-19 cases and hospital admissions in the UK was gathered from the UK Coronavirus Dashboard (1), which is updated every day. Data on the implementation of the COVID-19 certification in Scotland, England, Northern Ireland and Wales was collected from official sources, as mentioned in media (9–12). For all sources, we used data from the 1st of January 2021 to the 19th of January 2022. Data was extracted on the 19th of January 2022. Data from mid-year 2021 population for each country was extracted from the UK Coronavirus Dashboard (1) as well.

### COVID-19 certification

We studied the four countries of the UK (England, Scotland, Northern Ireland and Wales). A country was considered as implementing COVID-19 certification (CC) if the certificate was required for at least some frequently used public venues such as restaurants, nightclubs or cultural events. Scotland implemented COVID-19 certification on the 18th of October, Northern Ireland did it on the 29th of November. In the case of Wales, we modeled two different changes in the restriction of the certificate, as COVID-19 certification was first implemented for nightclubs on the 11th of October 2021 and then extended to cinemas, theaters and concert halls on the 15th of November 2021. England was the last country to require the certificate, only doing so after the 15th of December. See Table 1 for further detail on each country's implementation of the COVID-19 certification.

**Table 1:** Information on COVID-19 certification in all the countries of the UK.

**Region and intervention**	**Wales CC1**	**Wales CC2**	**Scotland CC1**	**Northern Ireland CC1**	**England CC1**
Intervention date	11/10/2021	15/11/2021	18/10/2021	29/11/2021	15/12/2021
Restrictions imposed	COVID-19 certificates—including either vaccination status or a negative COVID-19 test within the past 48 h—have been required to attend nightclubs, unseated indoor events with over 500 people, unseated outdoor events with over 4,000 people, and any event with over 10,000 people.	Extended to cinemas, theaters and concert halls.	COVID-19 certificates have been required to attend nightclubs, unseated indoor events with over 500 people, unseated outdoor events with over 4,000 people, and any event with over 10,000 people.	The pass would be mandatory in the same venues as Wales and also pubs and restaurants.	COVID-19 certificate will now be mandatory for nightclubs, unseated indoor events with 500 or more attendees, unseated outdoor events with 4,000 or more attendees and any event with 10,000 or more attendees.

### Outcomes

We studied two outcomes, for which we assessed the effect of the COVID-19 certification intervention: incidence rate of COVID-19 confirmed cases and incidence rate of COVID-19 hospital admissions in the general population. We introduced a lag after it to neglect data right after the intervention date, for which its effects were not expected to be significant. The lag was set to 5 days for COVID-19 cases and to 7 days for COVID-19 hospital admissions (13, 14).

### Study time intervals

We selected the time intervals for the study of each intervention as wide as possible, provided that they did not include more than one change in the intervention, that they included more than 10 points (days) at each side of the lag interval and that they did not show, if possible, exogenous changes in convexity.

### Models

We performed Negative binomial segmented regression (NB) and Autoregressive integrated moving average (ARIMA) models as a preliminary and sensitivity analysis of the main model, Difference-in-Differences. Detailed methods results and output for NB and ARIMA can be found in the Supplementary material.

Difference-in-Differences (DiD) methods compare the mean of the variable of interest for an exposed and control group, before and after a certain interruption point, providing insight on the changes of the variable for the exposed countries relative to the change in the negative outcome group (15). We cannot draw causal conclusions by simply observing before-and-after changes in outcomes, because other factors might influence the outcome over time. DiD methods overcome this by introducing a comparison between two similar groups exposed to different conditions. First, DiD takes the difference of the variable of interest of both groups before and after the intervention. Then it subtracts the difference of the control group to the difference of the exposed one to control for time varying factors, therefore giving a result which constitutes a difference of the differences. This approximates the clean impact of the intervention. In essence, the DiD estimating equation is the following,


$$\begin{array}{l} Y_{gt}=\beta_{0}+\beta_{1}T_{g}+\beta_{2}P_{t}+\beta_{3}\left(T_{g}xP_{t}\right)+\epsilon_{gt} \end{array}$$

where *Y*_*gt*_ is the outcome for an individual in group g and treated unit t, *P*_*t*_ is a binary time variable indicating whether the observation belongs to the period before or after the intervention and *T*_*g*_ is a binary variable indicating whether the observation belongs to the exposed or the controlled group. In this setting, the treatment effect is estimated with the coefficient β_3_ from the regression.

**Figure 1:**
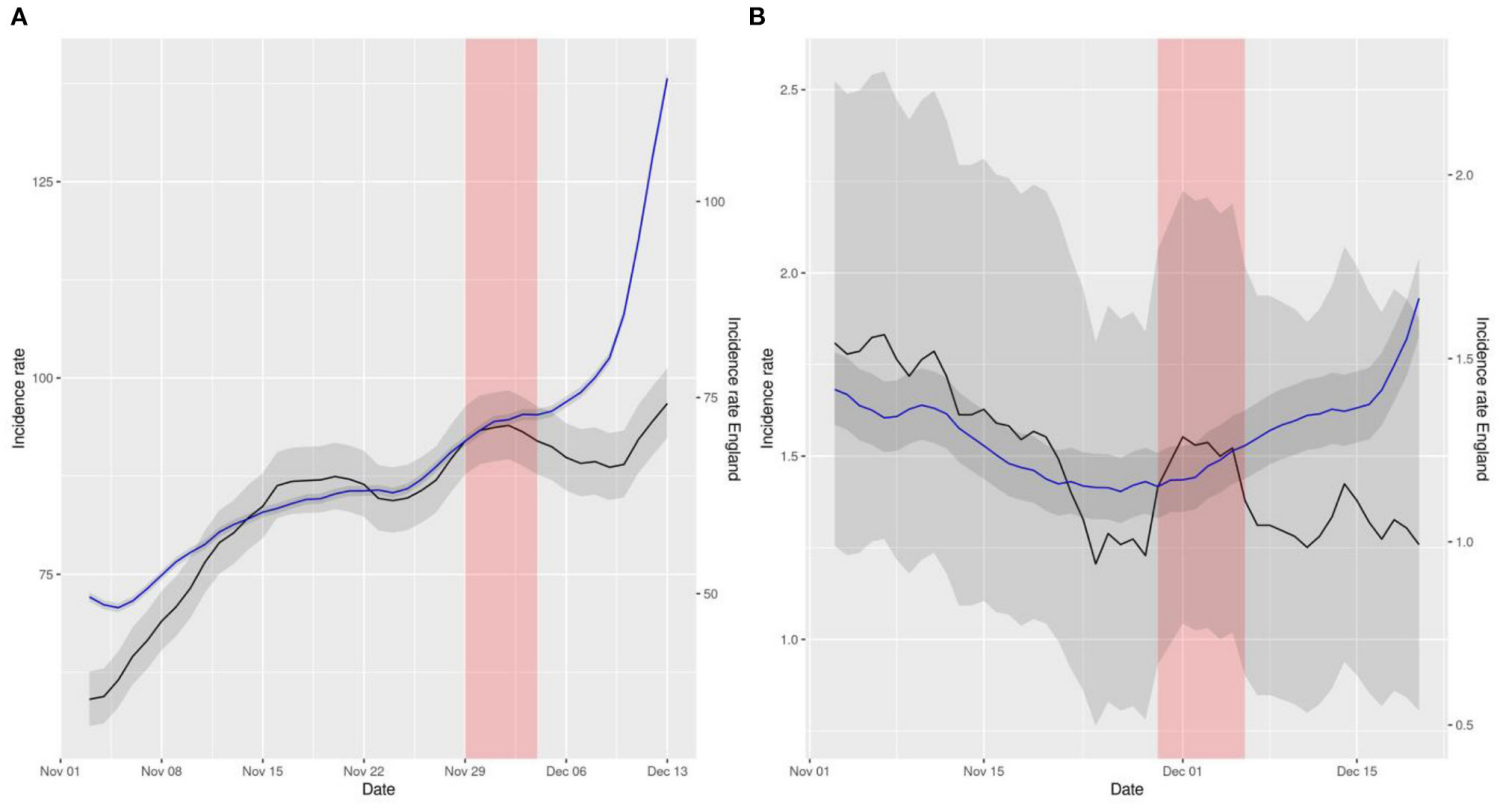
Representation of smoothed daily incidence rates of cases **(A)** and hospital admissions **(B)** in Northern Ireland (data in black) vs. England (data in blue) per 10^5^ inhabitants. Data has been displaced so that both curves intersect right at the intervention time point. The red shaded area represents the neglected period post-intervention in the model due to the lag between the intervention and its effect and the gray shaded area represents the 95% confidence intervals of the calculation of incidence rates.

**Figure 2:**
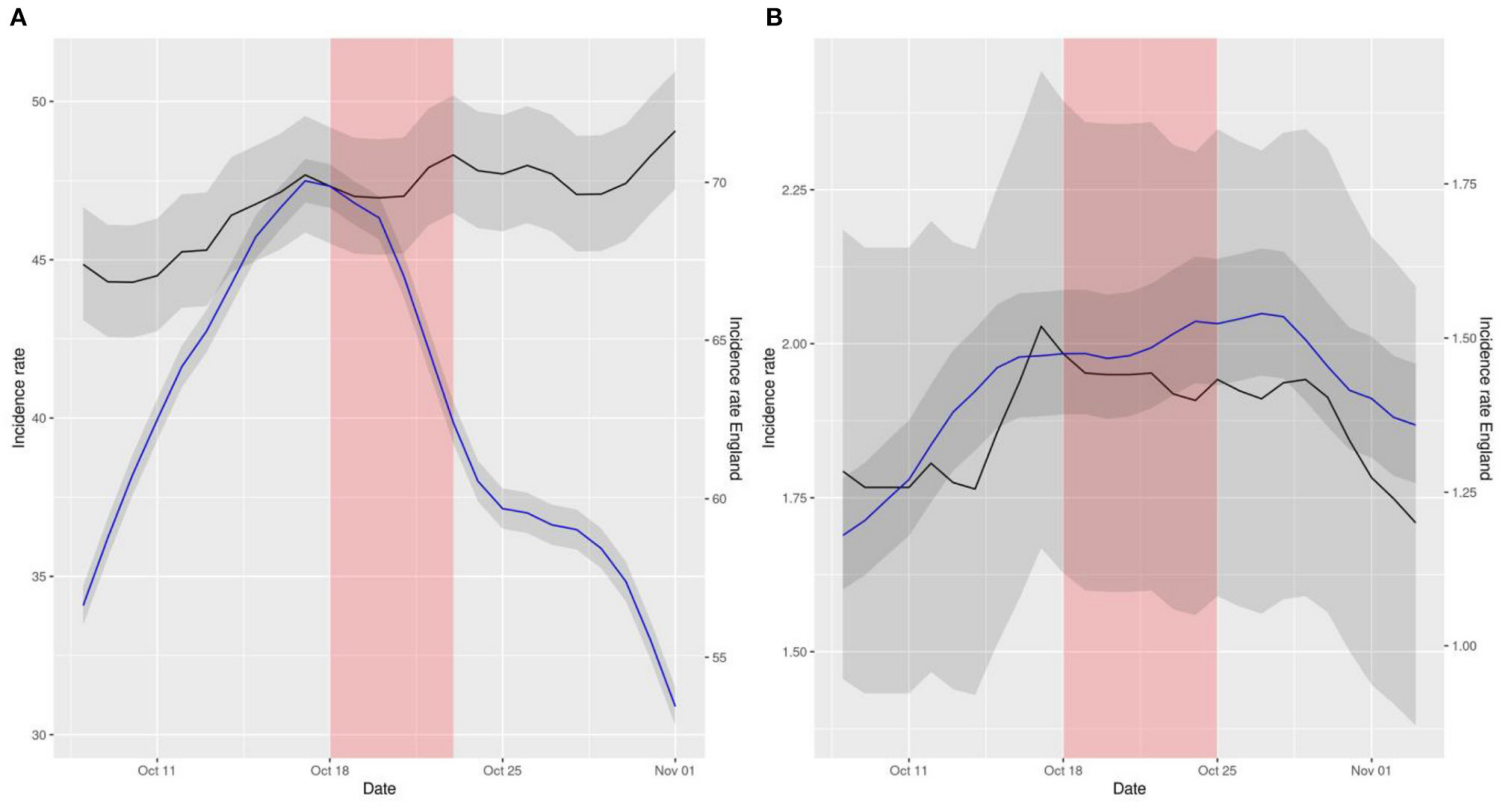
Representation of smoothed daily incidence rates of cases **(A)** and hospital admissions **(B)** in Scotland (data in black) vs. England (data in blue) per 10^5^ inhabitants. Data has been displaced so that both curves intersect right at the intervention time point. The red shaded area represents the neglected period post-intervention in the model due to the lag between the intervention and its effect and the gray shaded area represents the 95% confidence intervals of the calculation of incidence rates.

**Figure 3:**
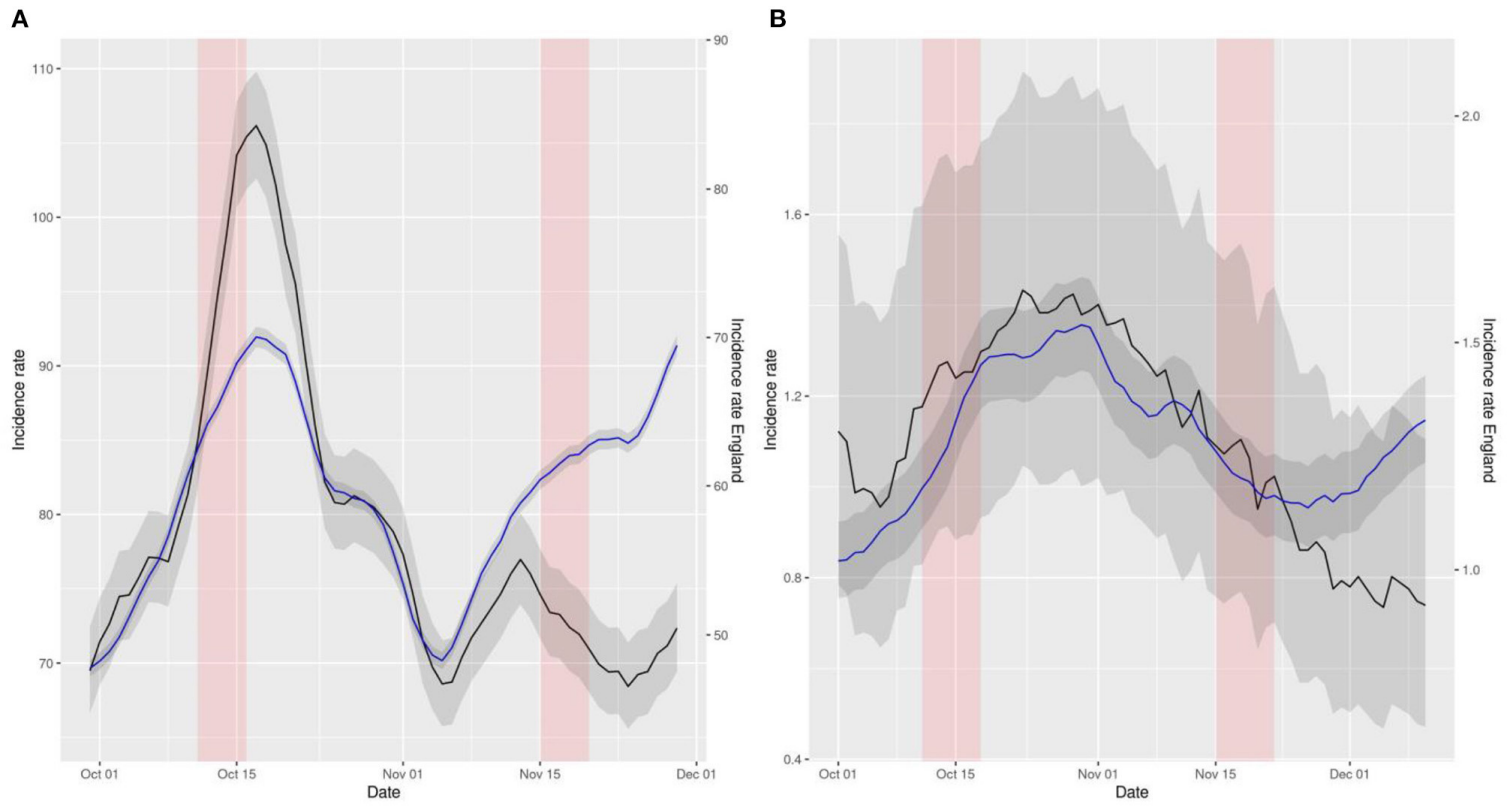
Representation of smoothed daily incidence rates of cases **(A)** and hospital admissions **(B)** in Wales (data in black) vs. England (data in blue) per 10^5^ inhabitants. Data has been displaced so that both curves intersect right at the intervention time point. The red shaded area represents the neglected period post-intervention in the model due to the lag between the intervention and its effect and the gray shaded area represents the 95% confidence intervals of the calculation of incidence rates.

For this method to be rightly used, all the typical OLS assumptions must be met. The parallel trends assumption, which requires both groups to present similar trends before the intervention time point (16), must also be satisfied. We tested all these assumptions, and the latter can be visually inspected in Figures 1–3.

DiD models produce estimates which consider a counterfactual group, therefore adjusting for unmeasured confounding. This cannot be done by neither of the two previous models. Its biggest limitation is that, in the end, the measured effect can only be attributed to the timepoint chosen. If that is due to the intervention placed then, or to other underlying reasons around the same time, cannot be known by design.

### Statistical analysis

We calculated incidence rates as number of cases (COVID-19 or admissions) divided by each country's population. We also calculated 7-day smoothed rolling average rates to reduce the effects of lower reporting on weekends.

We performed the first analysis on the 7-day smoothed data using NBSR. We also considered ARIMA models for autocorrelation.

To further strengthen the results and given that England did not implement the COVID-19 certification when it was effective in the other three countries, we used its data as a counterfactual for Difference-in-Differences (DiD) models. To help visualize this method, a plot of the difference and cumulative difference of the incidence rates for cases and hospitalizations of all countries is provided in Supplementary Figure 5. The numbers shown are essentially what constitute the basis of the DiD model. The differences have been calculated extracting England's incidence rates from the other countries' incidence rates. A decreasing trend in the difference's plots (on the left) is associated with a protective effect of the intervention date on the outcome.

We performed all the analyses in R v4.3 and used the packages epiR, tidyverse, forecast, ggplot2, MASS and lmtest. Code is available in https://github.com/KimLopezGuell/Covid-passport.

## Results

Table 2 contains results of a DiD regression for both cases and admissions incidence rates of the different UK countries (Wales, Northern Ireland, Scotland) compared to England. Except from cases in Scotland and cases in the first intervention in Wales, all the other COVID-19 certification introductions appeared to be effective against the spreading of the virus. Note the significance of the coefficients, in the sense that their 95% confidence interval does not include any positive subinterval.

**Table 2:** Estimates and 95% confidence intervals of the effect of the COVID-19 certification in the DiD models for the different countries and outcomes.

	**Wales CC1**	**Wales CC2**	**Northern Ireland CC1**	**Scotland CC1**
Cases IR estimate	2.2	−7.75	−10.1	7.91
95% CI cases	(−6.24,10.7)	(−13.1, −2.46)	(−18.4, −1.79)	(4.46, 11.4)
Admissions IR estimate	−0.144	−0.169	−0.269	−0.097
95% CI admissions	(−0.248, −0.039)	(−0.308, −0.031)	(−0.385, −0.153)	(−0.219, 0.024)

The numbers represent incidence rates per 1,00,000 people.

The reported coefficient of the DiD model is β_3_, which is the one we are most interested in. It is understood as how much the average outcome of the treatment group has changed in the period after the intervention, compared to what it would have happened to the same group had the intervention not occurred. Taking Wales CC1 admissions, −0.144 95% (95% CI −0.248, −0.039), this means that the incidence rate per 100,000 people was 0.144 units smaller on average in Wales after (and because of) the intervention.

The first COVID-19 certification introduction in Wales was not seen effective in terms of reduction on the number of cases, compared to England, with an associated coefficient of 2.22 (95% CI −6.24,10.7). It was associated, however, with a reduction of admissions, with a coefficient of−0.144 (95% CI −0.248,−0.039). In November, the increased restriction of the COVID-19 certification led to a decrease in the incidence rates of both outcomes compared to England, with coefficients −7.75 (95% CI −13.1, −2.46) for incidence rate of cases and −0.169 (95% CI −0.308, −0.031) for incidence rate of hospital admissions.

Northern Ireland showed a similar result, with coefficients −10.1 (95% CI −18.4, −1.79) and −0.269 (95% CI −0.385, −0.153) for incidence rates of cases and hospital admissions respectively.

As for the number of cases in Scotland, there also seemed not to be an effect of the COVID-19 certification, with a coefficient of 7.91 (95% CI 4.46, 11.4). Nonetheless the method indicated a significant effect on the incidence rate of hospital admissions, with a DiD coefficient of −0.097 (95% CI −0.219, 0.024).

The aforementioned comparisons can be visually inspected in Figures 1–3. These figures represent the incidence rates calculated using the raw data of COVID-19 outcomes and baseline numbers of population in all the countries taken from the COVID Dashboard (1). The 95% confidence intervals were calculated assuming that the data were distributed according to a Poisson distribution, which is common practice (17). The plots have been displaced, in the sense that each line has its own y axis, to allow the reader to test the linear trend assumption in DiD better.

NBSR and ARIMA models (Supplementary material) support these findings.

## Discussion

Using NBSR modeling, we found COVID-19 certification interventions were associated with a decrease in the incidence of COVID-19 cases in all countries except England, and with a decrease in COVID-19 hospitalizations only in Scotland. ARIMA models, which control for autocorrelation of the observations, supported these findings. DiD analyses supported a causal effect of CCs to decrease incidence rates in most territories, using England as a counterfactual. However, the study of COVID-19 certification intervention in England itself on the 15th of December 2021 shows that it was insufficient to prevent the increase in either the incidence of cases or hospital admissions in the country.

This discrepancy between the effect of COVID-19 passports in England compared to the other countries might be due to the new Omicron variant of the virus [which represented the 75% of the population of the country by that date (18)], the effect of other coexistent measures (like the mandatory use of face masks or accelerated booster vaccine campaign) or the already high uptake in vaccination. Indeed, as of 12th December 2021, almost 9 in 10 individuals aged 12 and over had been vaccinated with at least one dose (42,561,679, 88.0%), more than 8 in 10 individuals aged 18 and over had been vaccinated with both doses (38,627,544, 86.9%) and more than 6 in 10 individuals aged 40 and over had received a booster or 3rd dose (18,128,105, 63.8%) (19).

The visual difference in the NBSR plots, with England as a negative control group, reinforced the previous conclusions. Plots depicting the situation in Wales, for instance, suggested a striking effect compared to England. The intervention was not associated with a reduction of hospitalizations for some countries, but even in those cases, comparing to England, the plots indicated an impact of COVID-19 certification on reducing the increasing trend of hospital admissions observed at the same time in the English NHS.

In the DiD analyses, we found a significant effect of COVID-19 certification interventions for both incidence rates of cases and hospital admissions in Northern Ireland and the second half of November in Wales, compared to England, where the restriction was not into effect. The impact was not significant for the incidence rate of cases in Scotland nor October in Wales (first CC intervention), yet it was for hospital admissions. In fact, during that period the number of cases did decrease abruptly in Wales after the introduction of the COVID-19 certification. However, as they also decreased in England, the intervention effect was not so obvious. As for Scotland, the difference in trends pre-intervention for both groups is too acute to be able to interpret this model in a sensible way, as the assumptions for its validity are surely violated. In that sense, the DiD plots provided in the results section for all regions and outcomes, compared to England, in which both trends have been superposed to better see its similarities and differences, serve as a check for the validity of this assumption. We note, as commented before, that this condition is arguably satisfied for all pairs except for cases in Scotland. Hence, we can conclude that the reported effects of the certification as an intervention that reduced the incidence of COVID-19 are reliable.

These results are coherent with previous reported increased vaccine uptake after COVID-19 certification implementation (7, 8). Indeed, apart from the obvious restriction of mobility, the introduction of the COVID-19 certification and a subsequent increase in vaccine uptake could account for a lowering in both incidence of COVID-19 cases and hospitalizations. Moreover, this would explain the inefficiency observed in controlling the Omicron variant, as recent studies have reported lower effectiveness of the vaccines against infection by this variant (20–22). Indeed, these suggest that the Omicron variant can evade the immune protection conferred by vaccines, thus limiting their effectiveness to minimize the risk of infection.

Limitations of our analyses include the aggregated nature of our data, therefore potentially limited by ecological fallacy. Time varying influential factors have possibly been controlled with DiD methods taking England as a negative control group, yet other differences between the regions might be prevalent and affect the spreading of the virus differently. Moreover, the interventions were introduced at different times and with different limitations, and the response of the population to them might have been different in different regions. An unquestionably fair comparison is thus impossible.

On another note, an anticipatory effect of the certification could also be possible and has not been accounted for. Individuals might have pre-emptively reacted to the intervention, therefore pushing any potential consequences earlier in time. If so, accounting for a certain lag before the intervention date could be reasonable. This sensitivity analysis was not done in this study, and available data does not seem to indicate this should be of concern to our analyses.

It is important to stress that we cannot assert with undeniable confidence that these effects are due to COVID-19 certification, for a variety of reasons, stated throughout this paper. Mainly, we cannot disentangle effects of other contemporary measures from the effect of COVID-19 certification with this model. It is likely this intervention is linked to an increase in vaccination uptake, which is related to a change in the studied outcomes. Mandatory COVID-19 certification might be therefore a good measure for governments to implement, together with other measures, especially in areas with less vaccine coverage. It could be effective in limited periods of time and populations to boost vaccination. However, it cannot substitute a universal vaccination campaign with specific public health interventions to ensure equitable access to vaccines.

In conclusion, we demonstrate that the introduction of mandatory certificates was effective in decreasing cases in all countries except in England. This could be explained by differences of concomitant measures, on baseline vaccination uptake or by the emergence of the Omicron variant. Mandatory certification is only one of many policy levers to control the pandemic, and a sensible reassessment of its efficacy should be made by the competent authorities.

## Data availability statement

Publicly available datasets were analyzed in this study. This data can be found here: https://coronavirus.data.gov.uk/ and https://github.com/KimLopezGuell/Covid-passport/blob/main/Covid_passport.r.

## Author contributions

KL-G is a MSc student in Mathematical Sciences at the University of Oxford. She contributed to the formal analysis, creating tables and figures, and writing the original draft of the paper. AP-U is a DPhil student in the Pharmacoepidemiology group at the Centre for Statistics in Medicine (CSM) (Oxford) and an MD. He helped with the conceptualization of the project and also contributed to the formal analysis of the data and reviewed and edited the final written paper. MC is a postdoctoral scholar in the Pharmacoepidemiology group at the Centre for Statistics in Medicine (CSM) (Oxford), who also helped with the editing and review of the paper. CP is an Associate professor at the Computational Biology and Complex Systems group at the Universitat Politècnica de Catalunya. She helped with the conceptualization and editing and review of the paper. JH is a Professor of Bioinformatics at the University of Oxford. He helped with the editing and review of the paper. DP-A is a Professor of Pharmaco-and Device Epidemiology at the Centre for Statistics in Medicine (CSM) (Oxford) and an MD. He contributed to the conceptualization, formal analysis, and editing and review of the paper. He is the guarantor of the article. All authors contributed to the article and approved the submitted version.
