# Supplementary material for: The impact of COVID-19 certification mandates on the number of cases of and hospitalizations with COVID-19 in the UK: A difference-in-differences analysis
Source: Front Public Health. 2023 Feb 24;11:1019223. doi: 10.3389/fpubh.2023.1019223 (PMC9998475; doi:10.3389/fpubh.2023.1019223)
Supplement: Supplementary file 1 [file Data_Sheet_1.PDF]

SUPPLEMENTARY MATERIAL

Negative Binomial Segmented Regression and ARIMA models

Models

Segmented regression models consider a timepoint of potential change in the outcome of interest and model the time series at the two sides of that timepoint separately[15]. Linear segmented regression is a common model which assumes that the distribution of both sides of the intervention is linear, thus fitting two different lines at each side of the intervention and comparing their trends. This model allows to test the significance of the differences in slope or step very neatly. In our case, we use Negative Binomial (NB) Segmented Regression. This means that instead of modelling our data as if having a linear behaviour, we use a NB model. NB, as Poisson, is usually used to model counts data[16]. Furthermore, NB allows the variance to be quite larger than the mean of the counts, which we would expect when considering COVID-19 infections and hospitalisations in the general population. The model assumes that the data are indeed following a NB distribution, that the log of the outcome is related to the covariates linearly and that all the points are independent of each other. This latter assumption might be the most delicate, as we would expect our data to be timewise locally correlated.

That is why Autoregressive integrated moving average (ARIMA) provides further insight, as it can also model correlation between points[17]. In essence, these models use previous observations of the outcome as covariates for subsequent observations. This allows us to take into account these time relationships between our covariates to model the trend of the time series. The biggest limitation is that interpretability of the coefficients becomes harder than in the NBSR model, as now previous points are also covariates in the model, so coefficients cannot be thought of in the usual way (the effect on the outcome with 1 unit change of the covariate, while the rest of them are kept constant). Still, the sign of the coefficient of interest can be roughly interpreted as a positive (sign +) or negative (sign -) effect on the outcome[18]. In addition to that, this model has more parameters to be chosen than NBSR, for how far back to consider correlation, which induces more complexity.

These models can be sensitive to some of the specifications of the study design. In particular, the selected intervention timepoint is very important. That is the reason why it was pre-specified only using reliable information on intervention start date (see section COVID-19 certification). As for the 7 day moving averages, these make the outcome plots smoother, which should not have a huge impact on the general trend but do induce more correlation which ARIMA is able to pick up. The 5-day or 7-day lags post intervention date have a bigger effect on the estimation, as points are neglected and have no weight in the estimates for the post-intervention period. However, generally the period considered post-intervention is wide enough so that the potential effect of those points, were they wrongly neglected, should not change the estimates substantially. Also, post-intervention lag is essential when considering COVID-19 infections or hospitalisations as outcomes.

Statistical Analysis

We performed the first analysis on the 7-day smoothed data using Negative Binomial Segmented Regression (NBSR). We selected the time point of the intervention as specified before. The method provides insight on both changes in level and trend of the variable of interest after the intervention, and also allows for predictions of outcomes had the intervention not been put into effect.

In NBSR we are interested in studying the regression coefficients, of which a negative value is indicative of a reduction effect of the variable on the response. The exponential of the coefficients is used to extract the changes in slope directly. We assess both a step change and in the slope before

and after the intervention. Note that, while the former indicates a local reduction, the slope change is much more interesting and accounts for a temporarily longer effect. For the ARIMA models, we considered their sign as commented in the previous section.

Results

Table S1 provides estimates and 95% confidence intervals for the slope (t1) before the intervention, the step change of the non-pharmaceutical intervention (NPI) after the lag time and the change in the slope (t2) after the lag time for all NBSR models. The results suggest that COVID-19 certification is associated with the reduction of cases for all the countries of the UK apart from England, as well as with the reduction of hospital admissions for Scotland. The results from NBSR are reinforced by the respective ARIMA models.

			Wales CC1	Wales CC2	Northern Ireland CC1	Scotland CC1	England CC1
Cases	NBSR	Estimate t1 (%)	1.01	1.01	1.01	1.01	1.01
		95% CI t1 (%)	(0.99,1.04)	(0.99,1.04)	(1.00,1.02)	(0.97,1.04)	(1.00,1.02)
		Estimate t2 (%)	0.97	0.98	0.99	0.99	1.02
		95% CI t2 (%)	(0.94,0.99)	(0.95,1.02)	(0.97,1.01)	(0.95,1.03)	(1.00,1.04)
		Estimate NPI (%)	1.25	0.82	0.87	0.96	1.76
		95% CI NPI (%)	(0.98,1.59)	(0.61,1.12)	(0.73,1.04)	(0.67,1.40)	(1.54,2.00)
	ARIMA	Estimate t1 (%)	-0.33	1.15	1.05	0.33	1.31
		95% CI t1 (%)	(-3.03,2.38)	(-0.65,3.06)	(0.55,1.55)	(-2.70,3.37)	(0.39,2.23)
		Estimate t2 (%)	-1.73	-1.62	-0.16	-2.30	4.07
		95% CI t2 (%)	(-4.61, 1.15)	(-4.35, 1.11)	(-3.05, 2.73)	(-5.46, 0.87)	(1.48, 6.67)
		Estimate NPI (%)	39.30	-10.60	-12.40	32.33	78.00
		95% CI NPI (%)	(7.85, 70.79)	(-31.31, 10.13)	(-30.25, 5.43)	(-0.37, 65.03)	(44.93, 111.04)
Admissions	NBSR	Estimate t1 (%)	1.00	0.99	0.98	1.01	1.00
		95% CI t1 (%)	(0.81,1.24)	(0.92,1.06)	(0.94,1.02)	(0.86,1.19)	(0.97,1.04)
		Estimate t2 (%)	1.00	1.00	1.02	0.97	1.01
		95% CI t2 (%)	(0.77,1.31)	(0.89,1.12)	(0.91,1.13)	(0.77,1.22)	(0.96,1.06)
		Estimate NPI (%)	1.22	0.89	1.17	0.96	1.89
		95% CI NPI (%)	(0.09,22.29)	(0.19,4.36)	(0.31,4.19)	(0.12,8.53)	(0.74,5.12)
	ARIMA	Estimate t1 (%)	0	-0.02	-0.03	-0.67	0
		95% CI t1 (%)	(-0.05,0.04)	(-0.03,0)	(-0.04,0)	(-4.07,2.74)	(-0.01,0.03)
		Estimate t2 (%)	0.02	0.02	0.01	-2.16	0.05
		95% CI t2 (%)	(-0.04, 0.07)	(-0.01, 0.04)	(-0.03, 0.06)	(-6.98, 2.65)	(0.02,0.09)
		Estimate NPI (%)	0.32	-0.11	0.35	34.33	1.14
		95% CI NPI (%)	(-0.25, 0.89)	(-0.39, 0.17)	(-0.19, 0.88)	(-11.20, 79.87)	(0.53,1.75)

Table S1: Estimates and 95% confidence intervals of the effect of the COVID-19 certification in the NBSR and ARIMA models for the different countries and outcomes. NBSR coefficients are exponentiated. Information on trend pre-intervention (t1) and trend (t2) and level (NPI) change estimates after intervention are provided. *CC+number* indicates the number-th COVID-19 certification intervention. Information on the interventions can be found in *Table 1.*

The result for cases in Wales CC1 for t2, which is the coefficient of the change in slope, is 0.97 (95% CI 0.94,0.99). This would be read as “for every unit increase in time (days), the number of cases of COVID-19 decreases by 3% compared to the trend pre-intervention”. Regarding ARIMA, as commented before, the actual interpretation of the coefficients is more complicated, but the sign provides inference in the general trend influenced by the covariates, and therefore for the same example in Wales the coefficient -1.73 (95% CI -4.61,1.15) reinforces the previous statement.

Supplementary Figure S1 suggests that the introduction of COVID-19 certification stopped the increase in incidence rate of cases observed in early December 2021 in Northern Ireland, while it peaked in England, where no COVID-19 certification restriction was introduced, during the same time. We observed a daily raise of 1%, estimate 1.01 (95% CI 1.00,1.02) before the introduction of COVID-19 certification, which changed to a decrease in 1%, slope to 0.99 (95% CI 0.97,1.01) afterwards. As for incidence rate of hospital admissions, Northern Ireland shows a decreasing trend

during November, with a daily increase of 2%, slope 0.98 (95% CI 0.94,1.02), similar to England. Yet while in the latter the incidence rate increased rapidly with the arrival of the Omicron variant in the UK, the increase in Northern Ireland was much more moderate. With the introduction of the COVID-19 certification in the country, the slope of the trend increased to a 1.02 (95% CI 0.91,1.13).

Regarding Scotland, in *Supplementary Figure S2* we note that the incidence rate of cases presented an initial increase of 1% per day, estimate 1.01 (95% CI 0.97,1.04), which decreased to a slope of 0.99 (95% CI 0.95,1.03) after the intervention. As for the incidence rate of hospital admissions, the initial increase was of 1% per day, slope 1.01 (95% CI 0.86,1.19) and we observed a slope after COVID-19 certification introduction of 0.97 (95% CI 0.77,1.22).

While COVID-19 certification was associated with a slowdown of the incidence of cases in Wales during the second half of November, that was not seen in England (*Supplementary Figure S3a*). For Wales the pre-intervention slope was 1.01 (95% CI 0.99,1.04), which decreased to 0.97 after the intervention (95% CI 0.94,0.99). Likewise, hospital admissions (*Supplementary Figure S3b*) increased in England while decreasing in Wales in the same period.

An assessment of England (*Supplementary Figure S4*) itself during its introduction of COVID-19 certification on the 15th of December 2021 provided an estimate of the increase pre-intervention of 1.01 (95% CI 1.00,1.02) and a change in the slope to 1.02 (95% CI 1.00,1.04) for the incidence rate of cases. As for hospital admissions, the increase before the intervention was of 1.00 (95% CI 0.97,1.04) which changed to 1.01 (95% CI -0.96,1.06).

Plots

Northern Ireland

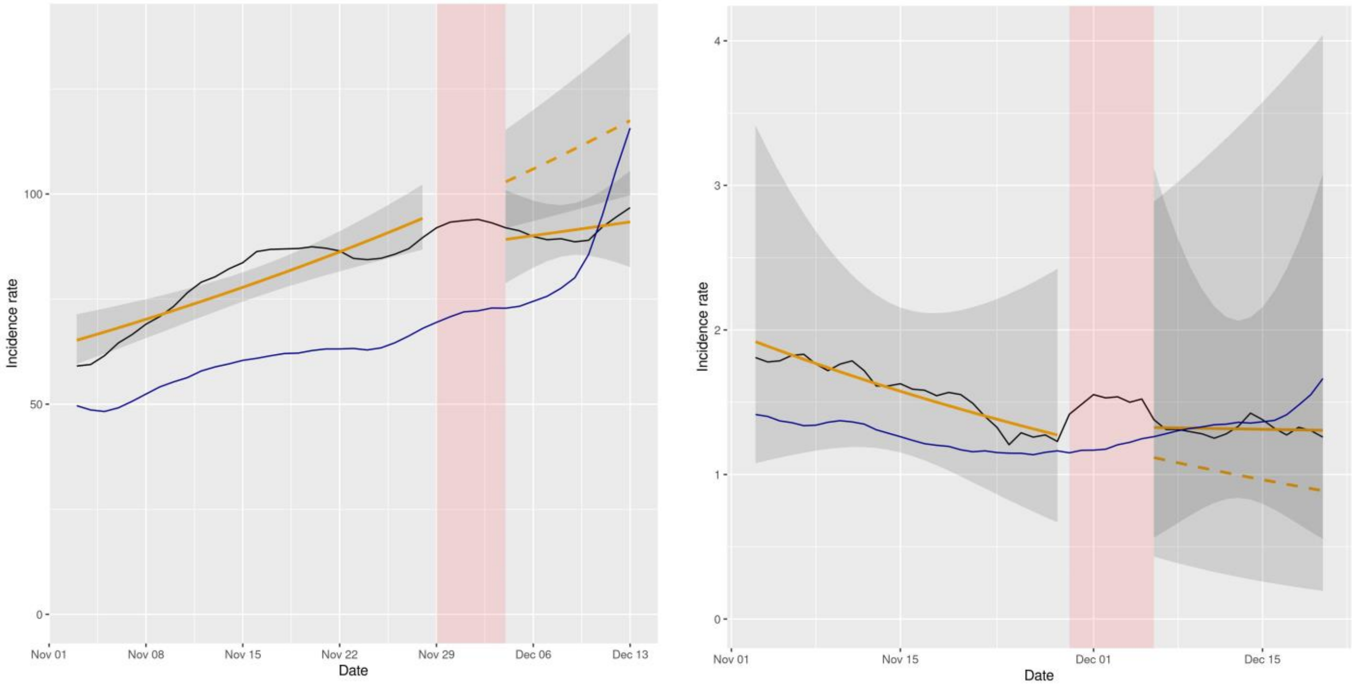

Figure S1a (left) and S1b (right). Representation of NBSR models comparing incidence rates of cases and hospital admissions in Northern Ireland (data in black, model in orange) vs England (data in blue). Dashed lines represent the expected evolution without intervention. The red shadowed area represents the neglected period post-intervention in the model due to the lag between the intervention and its effect and the grey shaded area represents the 95% confidence intervals for the predictions.

Wales

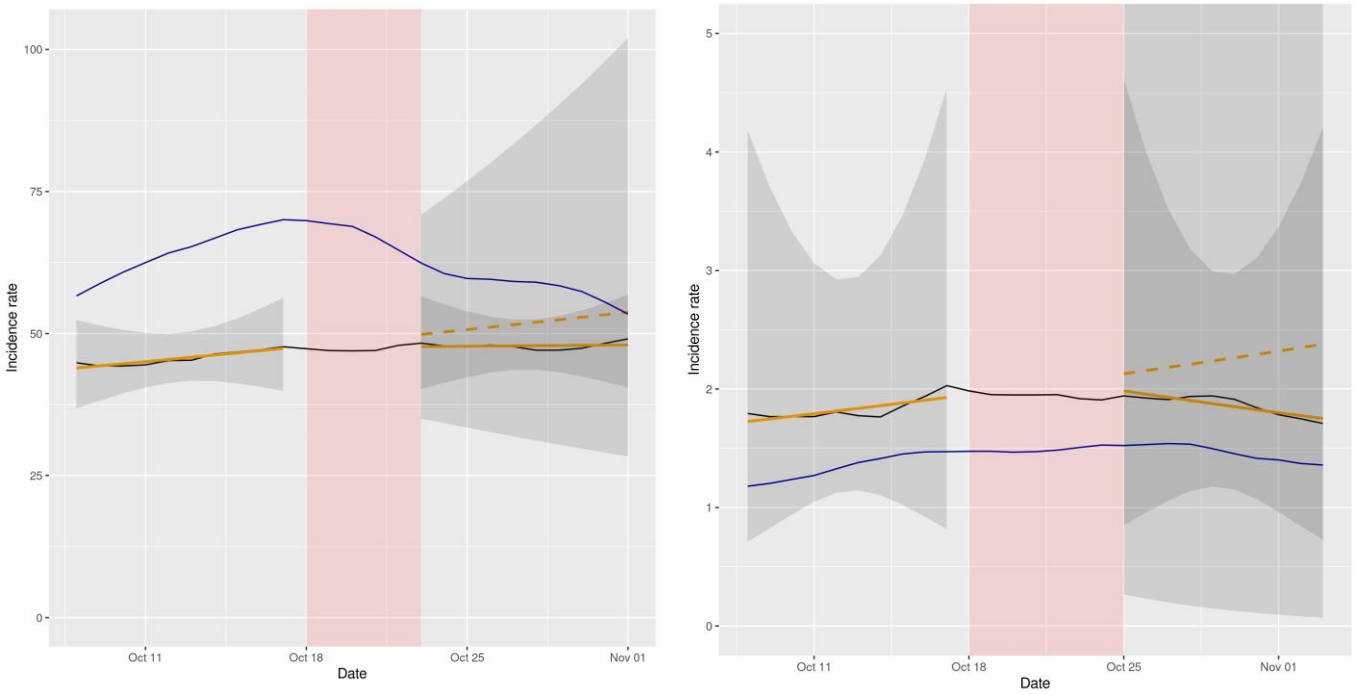

Figure S2a (left) and S2b (right). Representation of NBSR models comparing incidence rates of cases and hospital admissions in Scotland (data in black, model in orange) vs England (data in blue). Dashed lines represent the expected evolution without intervention. The red shadowed area represents the neglected period post-intervention in the model due to the lag between the intervention and its effect and the grey shaded area represents the 95% confidence intervals for the predictions.
Note: In figure 2b the y axis has been cut to make the trends more visible, as the confidence interval for the predictions post-intervention is relatively large.

Scotland

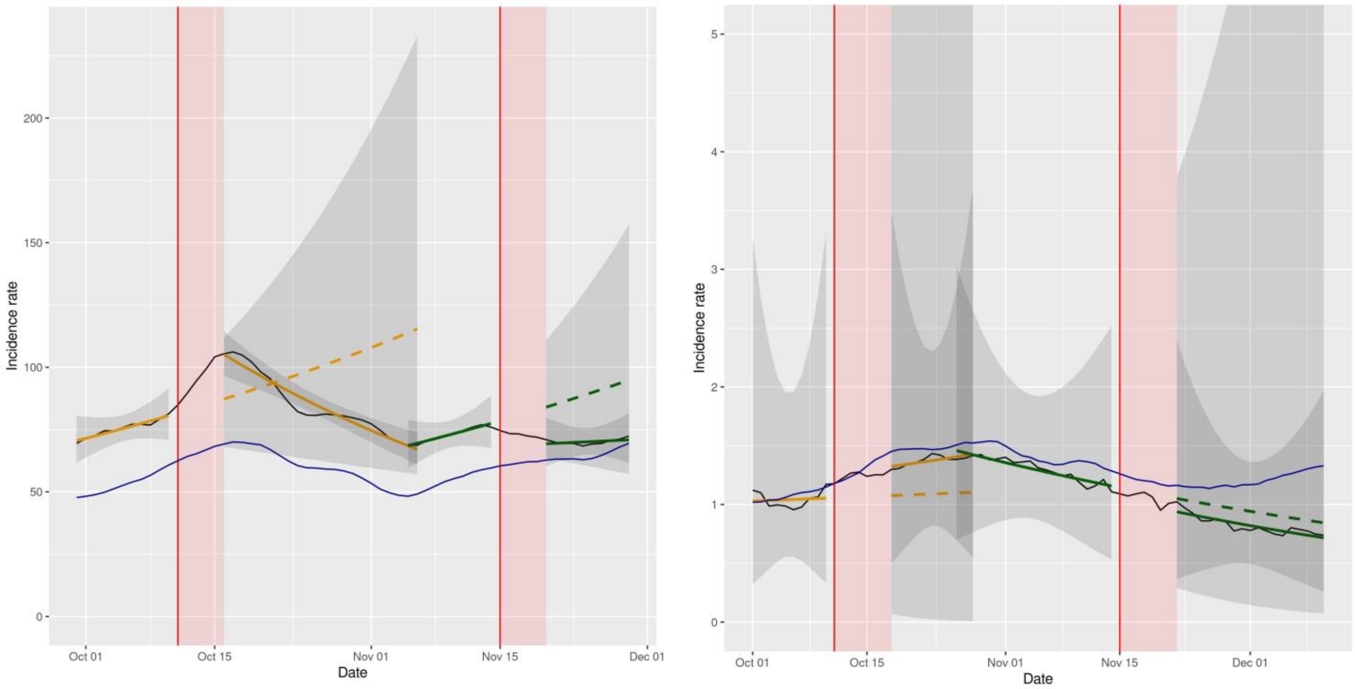

Figure S3a (left) and S3b (right). Representation of NBSR models comparing incidence rates of cases and hospital admissions in Wales (data in black, model in orange and in green) vs England (data in blue). Dashed lines represent the

expected evolution without intervention. The red shadowed area represents the neglected period post-intervention in the model due to the lag between the intervention and its effect and the grey shaded area represents the 95% confidence intervals for the predictions.

Note: In figure 3b the y axis has been cut to make the trends more visible, as the confidence interval for the predictions post-intervention is relatively large.

England

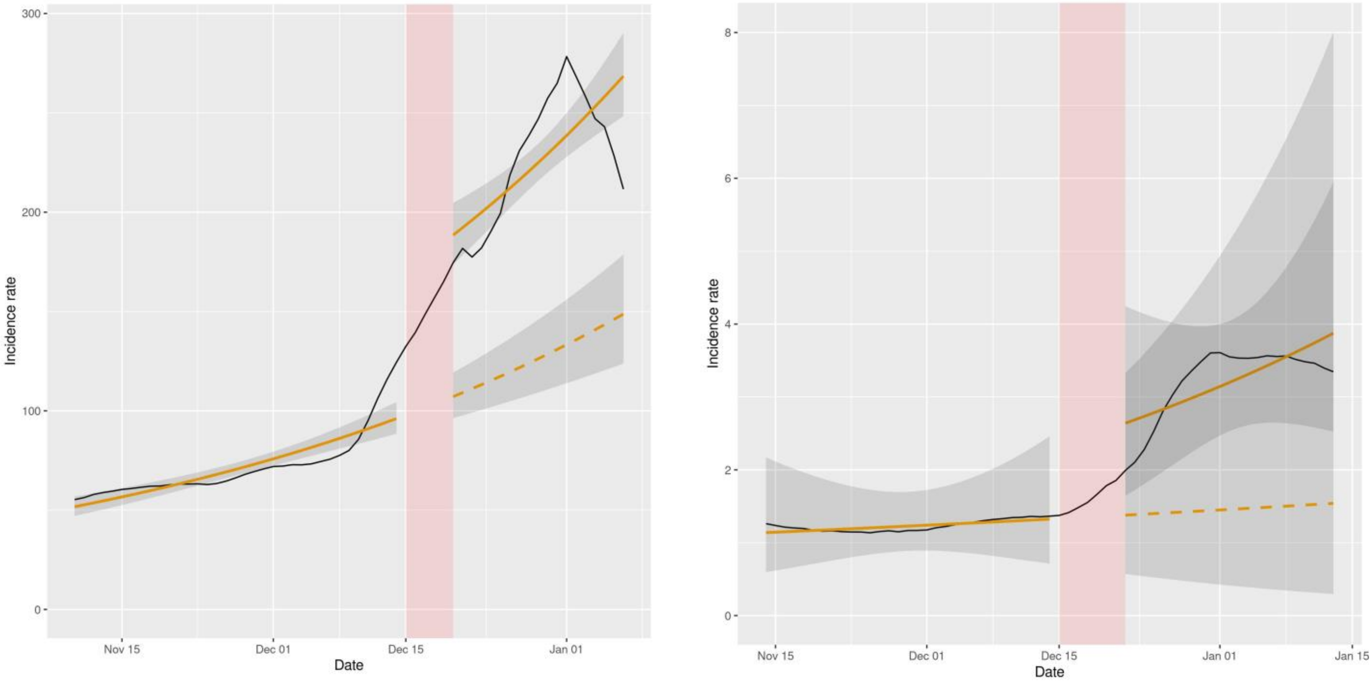

Figure S4a (left) and S4b (right). Representation of NBSR models of the incidence rates of cases and hospital admissions in England (data in black, model in orange). Dashed lines represent the expected evolution without intervention. The red shadowed area represents the neglected period post-intervention in the model due to the lag between the intervention and its effect and the grey shaded area represents the 95% confidence intervals for the predictions.
